# Determinants of COVID-19 vaccine hesitancy among students and parents in Sentinel Schools Network of Catalonia, Spain

**DOI:** 10.1371/journal.pone.0282871

**Published:** 2023-03-09

**Authors:** Fabiana Ganem, Cinta Folch, Andreu Colom-Cadena, Anna Bordas, Lucia Alonso, Antoni Soriano-Arandes, Jordi Casabona, on behalf of Sentinel School Network Study Group of Catalonia

**Affiliations:** 1 Centre d’Estudis Epidemiològics Sobre les ITS i Sida de Catalunya (CEEISCAT), Badalona, Spain; 2 Departament de Pediatria, d’Obstetrícia i Ginecologia i de Medicina Preventiva i de Salut Publica, Universitat Autònoma de Barcelona, Bellaterra, Spain; 3 Institut d’Investigació Germans Trias i Pujol (IGTP), Badalona, Spain; 4 Centro de Investigación Biomédica en Red de Epidemiología y Salud Pública (CIBERESP), Instituto de Salud Carlos III, Madrid, Spain; 5 Fundació Lluita Contra les Infeccions, Badalona, Spain; 6 Paediatric Infectious Diseases and Immunodeficiencies Unit, Hospital Universitari Vall d’Hebron, Barcelona, Spain; Universitas Syiah Kuala, INDONESIA

## Abstract

Vaccine hesitancy is defined as a delay in acceptance of vaccines despite its availability, caused by many determinants. Our study presents the key reasons, determinants and characteristics associated with COVID-19 vaccine acceptability among students over 16 years and parents of students under 16 years and describe the COVID-19 vaccination among students in the settings of sentinel schools of Catalonia, Spain. This is a cross-sectional study that includes 3,383 students and the parents between October 2021 and January 2022. We describe the student’s vaccination status and proceed a univariate and multivariate analysis using a Deletion Substitution Addition (DSA) machine learning algorithm. Vaccination against COVID-19 reached 70.8% in students under 16 years and 95.8% in students over 16 years at the end of the study project. The acceptability among unvaccinated students was 40.9% and 20.8% in October and January, respectively, and among parents was proportionally higher among students aged 5–11 (70.2%) in October and aged 3–4 (47.8%) in January. The key reason to not vaccinate themselves, or their children, were concern about side effects, insufficient research about the effect of the vaccine in children, rapid development of vaccines, necessity for more information and previous infection by SARS-CoV-2. Several variables were associated with refusal end hesitancy. For students, the main ones were risk perception and use of alternative therapies. For parents, the age of students, sociodemographic variables, socioeconomic impact related to the pandemic, and use of alternative therapies were more evident. Monitoring vaccine acceptance and refusal among children and their parents has been important to understand the interaction between different multilevel determinants and we hope it will be useful to improve public health strategies for future interventions in this population.

## Introduction

During the Coronavirus disease (COVID-19) emergency caused by Severe Acute Respiratory Syndrome Coronavirus 2 (SARS-CoV-2), different public health measures were implemented across the world, especially when the development of a vaccine was still quite immature. Currently, in a different scenario, several countries have been cooperating for the rapid development, update and distribution of vaccines, and the raised concerns has become the impact on vaccine effectiveness, considering the SARS-CoV-2 variants, like Omicron and its sub-lineages, able to reinfect people with previous infection or even fully vaccinated against COVID-19 [1, 2], as well as to guarantee their equitable distribution, acceptability [3, 4] assessing the barriers to effectiveness of vaccination programs, such as vaccine hesitancy [5].

On December 21, 2020, the European Commission authorized the first vaccine against SARS-CoV-2, the Comirnaty Pfizer-BioNTec for people over 18 years, after the positive opinion of the European Medicines Agency (EMA). On December 27, 2020, the vaccination started in Spain prioritizing specific and vulnerable groups such health care workers (HCW), school staff, older and people with underlying diseases [6].

In May 2021, EMA recommended an extension for the Comirnaty COVID-19 vaccine to 12 to 15 years and in November 2021 for children aged 5 to 11 years making it available for these groups [7].

Catalonia, a Northeast region of Spain with 7.7 million inhabitants, has registered 2.6 million SARS-CoV-2 infections, 118 thousand hospitalizations and 28 thousand deaths since it’s their first confirmed case on February 25, 2020. Vaccination coverage in Catalonia until November 2022 has reached 86.5% in people over 12 years, 79.5% in the 12–19 years and 33.7% in 5–11 [8].

Vaccine hesitancy was defined by the Strategic Advisory Group of Experts on Immunization (SAGE) as a “delay in acceptance or refusal of vaccines despite availability of vaccination services” that can be influenced by many factors such as the lack of offer, communication and confidence [9] and it is commonly observed with new vaccine or vaccine candidate [5]. There is mounting evidence showing that vaccination can promote an important control on burden and mortality caused by infectious diseases [10–12]. Therefore, vaccination is a cost-benefit intervention, being hesitation and vaccine refusal an important concern to public health.

Several studies were estimated the vaccine hesitancy among different populations. In June 2021, the prevalence of COVID-19 vaccine hesitancy in Spain was estimated at 6.22%, the lowest eight Europeans countries studied [11].

Despite differences between countries, significantly associations between vaccine hesitancy and sociodemographic determinants [13] (Hassan et al. 2021), vaccine confidence and distrust, misinformation, beliefs towards COVID-19 vaccination have already been observed in others studies [5]. A study that enrolled Asian, African and South America countries, founded that age, income, religion, comorbidities, economic situation during the COVID-19 pandemic and adherence to vaccination benefits were associated with COVID-19 vaccine hesitance [14], while in a study with European countries, gender, age, employment status, educational levels and vaccine skepticism, risk perception and compliance of public health measures and were associated with vaccine hesitance [11].

Epidemiological studies assessing determinants associated with the intention to vaccinate against SARS-CoV-2 may help to guide strategies for achieving the coverage that is necessary to avoid severity and mortality by COVID-19, and that could prevent the resurgence of this vaccine preventable disease [9, 11, 15–18].

Regarding the increasing burden of disease among children and young people, evidence suggests that the risk of long-term negative effects of COVID-19 in children is greater than the potential risks associated with vaccination against COVID-19 [19–21], immunization strategies against COVID-19 for children and adolescents must emphasize individual risks and benefits, recognizing for example, the emergence of more transmissible variants, such as Omicron [22, 23]. There is a debate about the real benefit-risk of COVID-19 vaccines in children/adolescents, due to a lower susceptibility to infection or COVID-19 observed in this population when compared with adults, the recommendation to vaccinate everyone aged 12 and over against COVID-19 has established itself as a critical strategy to control the pandemic [21].

This study is part of the COVID-19 Sentinel Schools Network of Catalonia (CSSNC) project, whose main objective are to monitor actively SARS-CoV-2 infections and identify barriers and facilitators for SARS-CoV-2 prevention strategies in schools. The CSSNC also provides an active surveillance during the pandemic that gathered evidence for enhanced of health protocols to prevent the spread of SARS-CoV-2 and others respiratory virus in the school [24].

The objectives of this study are to describe the COVID-19 vaccine acceptance, intentions and the key reasons for hesitation and refusal among students over 16 years and parents of students under 16 years, in the CSSNC, during the academic year 2021–2022, as well as to identify potential multilevel determinants for these parameters.

## Materials and methods

### Study design and population

This cross-sectional study was conducted in two different periods, October 2021 and on January 2022, in a setting of 23 schools participating in the CSSNC. In the first endpoint 3,383 students were included, aged 3–4 years (n = 213); 5–11 years (n = 1085); 12–15 years (n = 860) and 16–18 years (n = 1,225). In January 2022 there were, 2,635 students, aged 3–4 years (n = 196); 5–11 years (n = 1,035); 12–15 years (n = 834) and over16 years (n = 570 over).

### Data collection

Data collection was carried out in two points of time, the first in October 2021 and the second in January 2022. We collected nominal data through an online questionnaire, but a paper version was provided when necessary, and the questionnaires were also available in Catalan, Spanish and English. Two survey models were developed, one for students under 16 years, and another for students over 16 years.

The questionnaire contained questions about socioeconomic and demographic characteristics, behavior, compliance with preventive measures, impacts of the pandemic and vaccination status, and was adapted from the COSMO study survey [25].

For unvaccinated participants we asked if they would accept to receive the COVID-19 vaccine in the following months, whose response options were *accept*, *refuse and don’t know*. For those who expressed hesitancy and refusal intentions, we also asked about the reason.

The online questionnaire was prepared using REDCap, we shared it through a link with the school management team so that it could be sent to all students, parents or guardians and school staff. In addition, information panels were placed in schools, which included access to the survey via QR code. Before completing the survey, participants had to sign informed consent either in online or paper formats. In the second data collection, people who were already part of the project received the survey in their informed email.

The field team consisted of health professionals and researchers. Before starting the fieldwork, a series of sensitization meetings were held to inform the school community about the objectives of the study. In each school where participants were recruited, online and face-to-face meetings were held about the study with the participation of the project team and the educational community (families, teachers and school staff).

All participants under 16 years were guided by their parents or guardians, who answered the questionnaire and signed the informed consent. Students over 16 years answered the questionnaire and signed the informed consent by themselves. For this reason, we present the outcomes for the following groups: students older than 16 years (vaccination status and intentions), students younger than 16 years (vaccination status) and parents of students younger than 16 years (vaccination intentions).

### Outcomes and case definition

Our first outcome was vaccination status, as *vaccinated*, we considered those with at least one dose of COVID-19 vaccine and *unvaccinated*, individuals with no dose.

Regarding the vaccine intention, *vaccine acceptance* was used to describe a participant vaccinated with at least one dose of the COVID-19 vaccine or unvaccinated but with the intention of being vaccinated soon. We used *vaccine hesitancy* to describe unvaccinated participants, who were unsure whether they would be vaccinated and *vaccine refusal* to refers to all unvaccinated participants who expressed total refusal to be vaccinated.

Our second was outcome *vaccine acceptability*, defined as “Yes” if a participant was vaccinated with at least one dose of the COVID-19 vaccine or unvaccinated but with the intention of being vaccinated soon (*vaccine acceptance)* and as “No” for unvaccinated participants who were unsure whether they would be vaccinated (*vaccine hesitancy)* or unvaccinated participants who expressed total refusal to be vaccinated (*vaccine refusal)*.

### Independent variables

We investigated the association between characteristics and factors that could influence the intention of adolescents to get vaccinated and of parents to allow their children to be vaccinated against SARS-CoV-2. These factors were referred as independent variables including the following categories: sociodemographic, health, knowledge, attitudes and practices, measures to avoid a SARS-CoV-2 infection and COVID-19 related impacts. Each variable was categorized according to the question asked in the questionnaire (Table 1).

**Table 1:** Independent variables included in the study, CSSNC Catalonia, Spain 2021–2022.

**Sociodemographic**	
Sex	Male / Female
Age *	Grouped: 3–4 / 5–11 / 12–15 years / ≥16 years
Student and parents’ immigration status (birthplace)	Dichotomized: Spain / outside Spain (another countries)
Parents’ employment situation	Dichotomized: Employed / unemployed, retired and lay-of
Parents’ educational level	High education (university) / Secondary school / None or primary school
House size (in meters)	Dichotomized: >70m2 / <70m2
Residence size—People living in the same residence	Dichotomized: 1–4 / 5–9
Live with small/underage children	yes / no
Living with people over 65 years old	yes / no
Living with a healthcare work (HCW)	yes / no
**Health**	
Perception about current health status	Dichotomized: Bad (bad and fair) / Good (good, very good and excellent)
Perception about current mental health	Dichotomized: Bad (bad and fair) / Good (good, very good and excellent)
Have a chronic illness	yes / no
Previous COVID-19	No (No, and I had a negative test, I don’t know) / Yes (and I had a positive test)
**Knowledge, attitudes and practices**	
Perceived risk to become infected with SARS-CoV-2	Likert scale Dichotomized: unlikely (1–4) / very likely (5–7)
Perceived severity if–get infected with SARS-CoV-2	Likert scale Dichotomized: little severity (1–4) / very severity (5–7)
Avoid a SARS-CoV-2 infection during the pandemic is	Likert scale Dichotomized difficult (1–4) / easy (5–7)
Avoid SARS-CoV-2 infection in children during the pandemic	Likert scale Dichotomized Difficult (1–4) / easy (5–7)
Avoided contact with vulnerable people	Dichotomized Yes (most of the time and always) / No (sometimes, almost never and no)
Self-perceived knowledge about COVID-19 (a)	Likert scale Dichotomized: low (1–4) / high (5–7)
Factual knowledge about COVID-19 (a) (b)	low / high
**Measures and behavior to avoid a SARS-CoV-2 infection**	
Adherence to vaccination strategies is important	yes / no
Routine vaccination behavior is important	yes / no
Preventive Behavior in the last seven days	yes / no
Use of herbal supplements or homeopathies to prevent SARS-CoV-2 infection	yes / no
Use of antibiotics to prevent SARS-CoV-2 infection	yes / no
**COVID-19 related impacts**	
One of the parents lost his job during the pandemic	yes / no
One of the parents started working during the pandemic	yes / no
Family’s economic situation during the pandemic	Same / increase / decrease
Health status worsened during the pandemic overall	No (It has improved or remained the same) Yes (it has worsened)
Mental health worsened during the pandemic overall	No (It has improved or remained the same) Yes (it has worsened)

* Adjustment variable for participants under 16 years old

(a) Question asked only for students over 16 years

(b) Composite indicator created to measure knowledge about COVID-19 among students over 16 years. Three aspects, groups at risk, symptoms and means of transmission were evaluated through 21 questions. A point was assigned to each question, which at the end were added to classify the participant.

### Statistical analysis

A descriptive approach was carried out to present the vaccination status for all students and acceptability and reasons for refusal and hesitancy for parents of students under 16 years and students over 16 years. To describe the frequency of these variables, we present the data collected in October 2021 and January 2022. For the analysis, the student’s data were aggregated in two groups, under 16 years (3–15 years), and over 16 years due to different methodology of data collection.

Each variable had between 10 and 30% missing throughout the sample, resulting in missed observations, so prior to analysis, we performed an imputation of multiple data sets by the chained equation method, obtaining 20 imputed datasets [26], that allowed proceed univariate and multivariate analyzes with the same participants. We compared the values of imputed and unimputed variables using a chi-square test on the first set of imputed data, for each variable. We repeat the description presented in Tables 4 and 5, using the imputed dataset and, for univariate, we use the set of all imputed datasets. The univariate analysis was adjusted for a 3–15-year age group, using a binomial logistic regression, pooling the results through Rubin’s Rules (RR). We calculated the Odds Ratio (OR) with 95% confidence interval (CI) and p-value, which we considered p<0.05 statistically significant.

A multivariate analysis was performed only with the parents’ database (students under 16 year) due to the low number of unvaccinated students over 16 years (n = 24). We tested multicollinearity with a correlation matrix for each variable, excluding those with a correlation coefficient greater than 0.8 in absolute value.

In the multivariate model we proceed a Deletion Substitution Addition (DSA), with 50 iterations. DSA is an algorithm for Machine Learning that chooses the model with the smallest Root Mean Square Error (RMSE). This method consists of adding, removing and replacing variables and performing a cross-validation in 5 rounds with 5 iterations, resampling the database in 5 random partitions where each model iterations is done in one partition and validated in the other 4, making that the model to be tested not only on the main sample but also on different subsamples. The DSA function was run in one imputed dataset, and a logistic regression was performed to all imputed datasets to keep only the significant variables, through a backward elimination. To ensure consistent results, the DSA model was used on the first and last imputed datasets, and on one more randomly selected dataset (dataset 11), and as a result, we obtained the same final model. The performance of the models was accessed by the Akaike Information Criterion (AIC), adjusted R-square, criteria that defined the choice for the DSA model.

We performed a McNemar test, which selects participants who were matched in the two samples (N = 2,145), to see if there was significance between the difference in acceptability rates between October 2021 and January 2022 between parents and students.

All analyses were carried out with R (version 4.1.2). The imputation was proceeded by *mice* package and the Rubin’s Rules by the *pool* function in the same package, in RStudio Software [27].

## Ethics approval

This study was approved on 17 December 2020 by the Ethical Committee of the Foundation University Institute for Research in Primary Health Care Jordi Gol i Gurina (IDIAPJGol) (code 20/192-PCV). A written informed consent was obtained from school staff, and since it includes minors, was signed for parents or guardians of children under 16 years and by participants older than 16 years that signed by themselves. All documents were archived in a place accessed only by the researchers. The signed document provided information about, procedures, risks, use of the collected data, anonymity, confidentiality, and general information about the study. All participants were free to decline/withdraw consent at any time without providing a reason and without being subject to any resulting detriment.

## Results

### COVID-19 vaccination status and intentions

The vaccination coverage against SARS-CoV-2 in CSSNC was 36.1% for students under 16 years and 92.4% for students over 16 years in October 2021, increasing to 70.8% and 95.8% <16y-old and >16y-old, respectively, in January 2022. Among unvaccinated students over 16 years 28 (30.1%) and 15 (62.5%), in October and January respectively, refused to be vaccinated. The acceptability among their parents was proportionally higher among students aged 5–11 in October (70.2%) and students aged 3–4 in January (47.8%) but considering the denominators this result must be interpreted with caution, the parents’ intention also varied significantly between October 2021 and January 2022, according to the McNemar test (Table 2).

**Table 2:** Vaccination against SARS-CoV-2 and intentions among unvaccinated students over 16 years and parents of unvaccinated students under 16 years at SSNC, Catalonia, Spain. October 2021 and January 2022.

	October 2021								January 2022							
	Age group (years)								Age group (years)							
	3–4		5–11		12–15		>16		3–4		5–11		12–15		>16	
	N = 213		N = 1085		N = 860		N = 1225		N = 196		N = 1035		N = 834		N = 570	
	n	%	n	%	n	%	n	%	N	%	n	%	n	%	n	%
**Vaccination Status**																
Unvaccinated	213	100%	1080	99.5%	88	10.2%	93	7.6%	184	93.9%	385	37.2%	33	4.0%	24	4.2%
Vaccinated	0	0%	5	0.5%	772	89.8%	1132	92.4%	12	6.1%	650	62.8%	801	96.0%	546	95.8%
**Intentions among unvaccinated**																
Accept	125	58.7%	756	70.2%	49	56.3%	38	40.9%	88	47.8%	161	41.8%	8	24.2%	5	20.8%
Hesitancy	71	33.3%	245	22.7%	26	29.9%	27	29.0%	67	36.4%	151	39.2%	9	27.3%	4	16.7%
Refusal	17	8.0%	76	7.1%	12	13.8%	28	30.1%	29	15.8%	73	19.0%	16	48.5%	15	62.5%

### Reasons and determinants to hesitancy and refusal of COVID-19 vaccine

**Fig 1:**
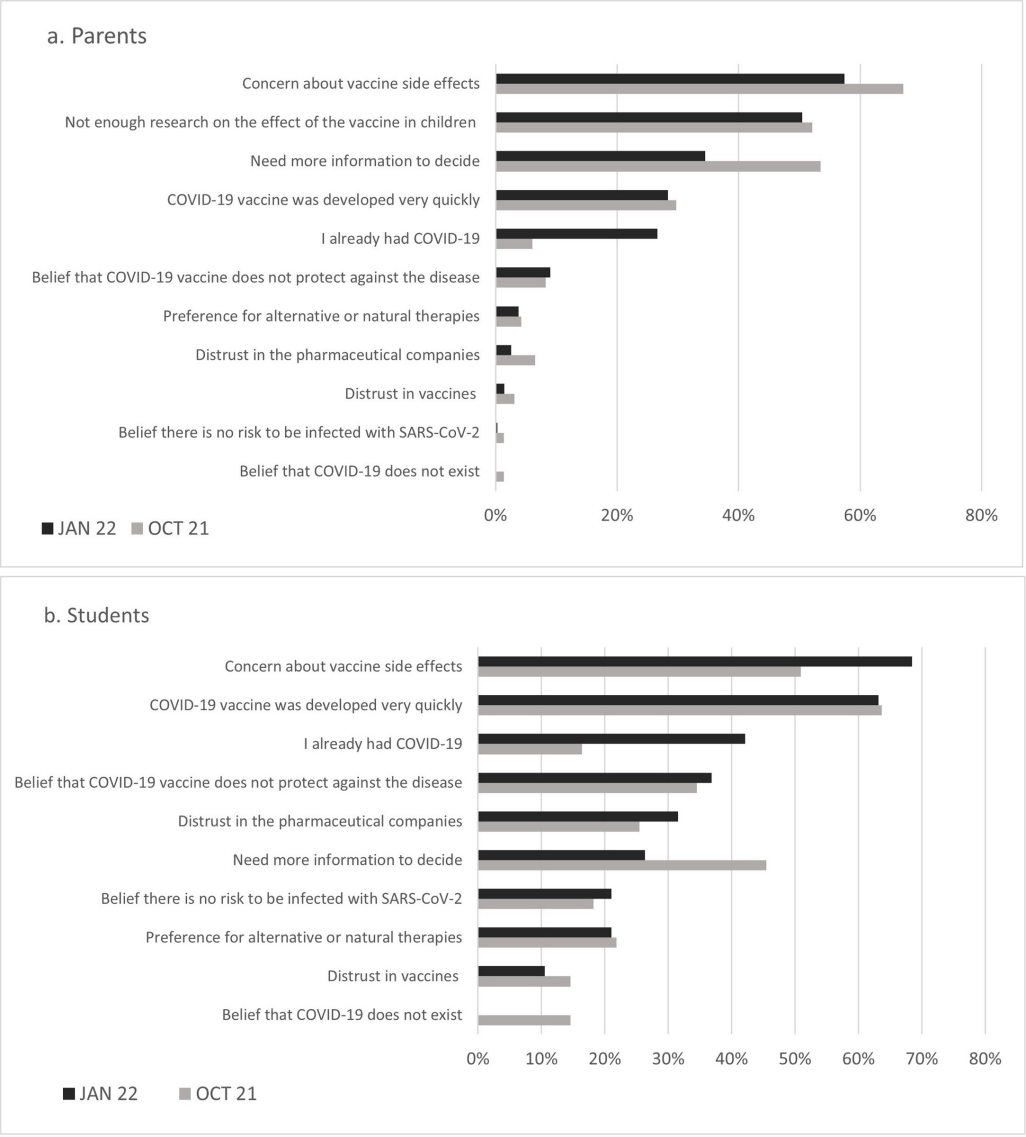
Reported reasons to SARS-CoV-2 vaccine hesitancy or refusal among unvaccinated. Fig 1A for parents of unvaccinated students under 16 years and Fig 1B for students over 16 years. The N for parents was 447 in October 2021 and 55 in January 2022 and for students was 345 in October 2021 and 19 in January 2022. SSNC project, Catalonia, Spain. October 2021 and January 2022.

We investigated the reasons for hesitation or refusal among parents and students in this study. The 11 key reasons informed by parents in both endpoints, were quite similar, *concerning about side effects* (67.1% and 57.4% respectively); *insufficient research about vaccine safety and efficacy in children* (52.1% and 50.4% respectively) and demand *for more information to deciding* to vaccinate their children (53.5% and 34.5% respectively). For students, the key reasons to hesitancy or refusal were slightly different in the two periods. In October 2021 most unvaccinated students reported *concern with the time to development the vaccine* (63.6%), *concern about side effects* (50.9%) and *necessity for more information before deciding to vaccinate* (45.5%). In January 2022, the main reason *was concerning about side effects* (68.4%), *time to development the vaccine* (63.2%) and *previous COVID-19 disease* (42.1%) (Fig 1A and 1B).

### Students’ descriptive and univariate analysis

The perception that it is easy to avoid a SARS-CoV-2 infection (OR 0.29 95%CI 0.09–0.88) and the use of herbal supplements and homeopathies to avoid a SARS-CoV-2 infection (OR 0.22 95%CI 0.08–0.63) were negatively associated with vaccine acceptance. A higher self-perceived knowledge (OR 3.6 95%CI 1.27–10.11) were positively associated with acceptability. As expected, adherence to vaccination strategies overall (OR 15.23 95%CI 5.13–45.19) and routine vaccination behavior (OR 5.49 95%CI 2.08–14.49) was strongly and positively associated with vaccine acceptability (Table 3).

**Table 3:** Descriptive and univariate analysis between sociodemographic, health status, perception, attitudes and knowledge, behavior, COVID-19 related impacts and vaccine acceptability among students over 16 years in SSNC. Catalonia, January 2022.

Variables	Acceptability		Univariate analysis*		
	No N = 19	Yes N = 551	OR	CI95%	p-value
**Sociodemographic**					
Sex					
Female	14 (3.57%)	378 (96.4%)			ref
Male	5 (2.81%)	173 (97.2%)	1.28	0.45–3.61	0.639
**Health**					
Perception about current health status					
Bad	0 (0.00%)	30 (100%)	-	-	-
Good	19 (3.52%)	521 (96.5%)	-	-	-
Perception about your current mental health					
Bad	8 (4.88%)	156 (95.1%)			ref
Good	11 (2.71%)	395 (97.3%)	1.4	0.51–3.86	0.514
Have a chronic illness					
No	19 (3.76%)	486 (96.2%)	-	-	-
Yes	0 (0.00%)	65 (100%)	-	-	-
Previous COVID-19					
No or don’t know	15 (3.38%)	429 (96.6%)			ref
Yes	4 (3.17%)	122 (96.8%)	0.87	0.27–2.82	0.822
**Knowledge, perceptions, attitudes and practices**					
Perceived risk to become infected with SARS-CoV-2					
Unlikely	18 (4.02%)	430 (96.0%)			ref
Very likely	1 (0.82%)	121 (99.2%)	3.93	0.51–29.96	0.188
Perceived severity if get infected with SARS-CoV-2					
Won’t be very sick	17 (3.91%)	418 (96.1%)			ref
Will be very sick	2 (1.48%)	133 (98.5%)	1.96	0.43–8.92	0.384
Avoid a SARS-CoV-2 infection during the pandemic is**					
Difficult	6 (1.99%)	296 (98.0%)			ref
Easy	13 (4.85%)	255 (95.1%)	0.29	0.09–0.88	0.029
Avoid SARS-CoV-2 infection in children during the pandemic					
Difficult	12 (3.06%)	380 (96.9%)			ref
Easy	7 (3.93%)	171 (96.1%)	0.79	0.29–2.17	0.649
Avoided contact with vulnerable people					
No	6 (3.82%)	151 (96.2%)			ref
Yes	13 (3.15%)	400 (96.9%)	1.2	0.41–3.54	0.739
Self-perceived knowledge about COVID-19**					
Low	12 (5.58%)	203 (94.4%)			ref
High	7 (1.97%)	348 (98.0%)	3.58	1.27–10.11	0.017
Factual knowledge about COVID-19					
Low	7 (5.83%)	113 (94.2%)			ref
High	12 (2.67%)	438 (97.3%)	2.49	0.93–6.67	0.069
**Measures and behavior to avoid a SARS-CoV-2 infection**					
Adherence to vaccination strategies is important**					
No	14 (15.4%)	77 (84.6%)			ref
Yes	5 (1.04%)	474 (99.0%)	15.23	5.13–45.19	<0.001
Routine vaccination behavior is important**					
No	11 (9.73%)	102 (90.3%)			ref
Yes	8 (1.75%)	449 (98.2%)	5.49	2.08–14.49	0.001
Preventive behavior to prevent SARS-CoV-2 infection (in the last seven days)					
No	3 (7.89%)	35 (92.1%)			ref
Yes	16 (3.01%)	516 (97.0%)	2.88	0.78–10.53	0.111
Use of herbal supplements or homeopathies as a health measure to prevent SARS-CoV-2 infection**					
No	11 (2.23%)	483 (97.8%)			ref
Yes	8 (10.5%)	68 (89.5%)	0.22	0.08–0.63	0.004
Used antibiotics as a health measure to prevent SARS-CoV-2 infection					
No	16 (3.43%)	450 (96.6%)			ref
Yes	3 (2.88%)	101 (97.1%)	0.97	0.27–3.39	0.956

* Univariate analysis adjusted by age for a 3–15-year age group, using a binomial logistic regression with all 20 imputed datasets, pooling the results through Rubin’s Rules (RR).

** Significative variables (p<0.050)

### Parents’ descriptive, univariate and multivariate analysis

A negative association was found between COVID-19 vaccine acceptability and birthplace, when at least one of the parents was born outside Spain (OR 0.63 95%CI 0.46–0.86), houses with more than five people living together (OR 0.7 95%CI 0.49–0.99), decrease of family’s economic situation during the pandemic (OR 0.65 95%CI 0.47–0.9), use of herbal supplement or homeopathy as a measure to prevent a SARS-CoV-2 infection (OR 0.44 95%CI 0.29–0.67), concern about spending too much time with family (OR 0.53 95%CI 0.29–0.97) (Table 4).

**Table 4:** Descriptive and univariate analysis between sociodemographic, health status, perception, attitudes and knowledge, behavior, COVID-19 related impacts and vaccine acceptability among parents of students under 16 years in SSNC. Catalonia, January 2022.

Variables	Acceptability		Univariate analysis*		
	No	Yes	OR	CI95%	p-value
	N = 345	N = 1720			
**Sociodemographic**					
Student age group					
3–5	132 (43.3%)	173 (56.7%)			
6–11	188 (20.3%)	738 (79.7%)			
12–15	25 (3.00%)	809 (97.0%)			
Sex					
Female	187 (17.5%)	883 (82.5%)			ref
Male	158 (15.9%)	837 (84.1%)	1.14	0.89–1.46	0.297
Students’ immigration status (birthplace)					
Spain	331 (16.6%)	1663 (83.4%)			
Another country	14 (19.7%)	57 (80.3%)	0.65	0.34–1.25	0.199
Parents immigration status (birthplace)**					
Spain	273 (15.8%)	1457 (84.2%)			ref
Another country	72 (21.5%)	263 (78.5%)	0.63	0.46–0.86	0.004
Father employment situation **					
Unemployed, retired or lay-of	33 (30.6%)	75 (69.4%)			ref
Employed	312 (15.9%)	1645 (84.1%)	2.45	1.46–4.1	0.001
Mother employment situation **					
Unemployed, retired or lay-of	64 (23.4%)	210 (76.6%)			ref
Employed	281 (15.7%)	1510 (84.3%)	1.68	1.14–2.47	0.009
Father educational level					
None or primary school	40 (12.0%)	292 (88.0%)			ref
secondary school	150 (19.2%)	631 (80.8%)	0.75	0.47–1.19	0.217
University	155 (16.3%)	797 (83.7%)	1.06	0.66–1.72	0.803
Mother educational level					
None or primary school	26 (15.4%)	143 (84.6%)			ref
secondary school	112 (17.9%)	513 (82.1%)	0.83	0.46–1.49	0.527
University	207 (16.3%)	1064 (83.7%)	1.1	0.64–1.88	0.736
House size (in meters)					
<70m2	62 (21.7%)	224 (78.3%)			ref
>70m2	283 (15.9%)	1496 (84.1%)	1.1	0.77–1.58	0.603
Number of people living in the same residence**					
1–4	273 (16.1%)	1426 (83.9%)			ref
5–9	72 (19.7%)	294 (80.3%)	0.7	0.49–0.99	0.046
Live with small/underage children					
No	105 (16.1%)	546 (83.9%)			ref
Yes	240 (17.0%)	1174 (83.0%)	1.1	0.82–1.47	0.539
Living with people over 65 years					
No	330 (16.6%)	1652 (83.4%)			ref
Yes	15 (18.1%)	68 (81.9%)	1.09	0.52–2.28	0.817
Live with a health care worker (HCW)**					
No	292 (17.2%)	1403 (82.8%)			ref
Yes	53 (14.3%)	317 (85.7%)	1.52	1.03–2.25	0.036
**Health status**					
Perception about current health status **					
Bad	5 (35.7%)	9 (64.3%)			ref
Good	340 (16.6%)	1711 (83.4%)	4.68	1.16–18.84	0.031
Perception about current mental health status					
Bad	9 (14.3%)	54 (85.7%)			ref
Good	336 (16.8%)	1666 (83.2%)	2.26	0.94–5.42	0.069
Have a chronic illness					
No	324 (16.8%)	1610 (83.2%)			ref
Yes	21 (16.0%)	110 (84.0%)	0.76	0.43–1.35	0.344
Previous COVID					
No or don’t know	289 (16.1%)	1510 (83.9%)			ref
Yes	56 (21.1%)	210 (78.9%)	0.69	0.47–1.01	0.058
**Perceptions, attitudes and practices**					
Perceived risk to become infected with SARS-CoV-2					
Unlikely	279 (16.3%)	1437 (83.7%)			ref
Very likely	66 (18.9%)	283 (81.1%)	1.02	0.7–1.48	0.91
Perceived severity if get infected with SARS-CoV-2					
Won’t be very sick	296 (17.0%)	1448 (83.0%)			ref
Will be very sick	49 (15.3%)	272 (84.7%)	1.08	0.72–1.61	0.705
Avoid SARS-CoV-2 infection in children during the pandemic					
Difficult	283 (17.0%)	1377 (83.0%)			ref
Easy	62 (15.3%)	343 (84.7%)	0.95	0.67–1.35	0.765
Avoided contact with vulnerable people					
No	92 (21.8%)	330 (78.2%)			ref
Yes	253 (15.4%)	1390 (84.6%)	1.3	0.95–1.79	0.099
**Measures and behavior to avoid a SARS-CoV-2 infection**					
Adherence to vaccination strategies is important**					
No	23 (57.5%)	17 (42.5%)			ref
Yes	322 (15.9%)	1703 (84.1%)	18.8	6.93–50.98	<0.001
Routine vaccination behavior is important					
No	9 (21.4%)	33 (78.6%)			ref
Yes	336 (16.6%)	1687 (83.4%)	2.7	1.08–6.75	0.034
Preventive behavior to prevent SARS-CoV-2 infection					
No	0 (0.00%)	15 (100%)	*	*	*
Yes	345 (16.8%)	1705 (83.2%)	*	*	*
Use of herbal supplements or homeopathies as a health measure to prevent SARS-CoV-2 infection **					
No	288 (15.3%)	1589 (84.7%)			ref
Yes	57 (30.3%)	131 (69.7%)	0.44	0.29–0.67	<0.001
Used antibiotics as a health measure to prevent SARS-CoV-2 infection					
No	329 (16.6%)	1655 (83.4%)			ref
Yes	16 (19.8%)	65 (80.2%)	0.71	0.36–1.4	0.319
**COVID-19 related impacts**					
One of the parents lost his job during the pandemic**					
No	324 (16.3%)	1664 (83.7%)			ref
Yes	21 (27.3%)	56 (72.7%)	0.53	0.29–0.97	0.038
One of the parents started working during the pandemic					
No	334 (16.5%)	1694 (83.5%)			ref
Yes	11 (29.7%)	26 (70.3%)	0.6	0.25–1.45	0.258
Family’s economic situation during the pandemic**					
Same	246 (15.2%)	1375 (84.8%)			ref
Decrease	85 (21.4%)	313 (78.6%)	0.65	0.47–0.9	0.01
Increase	14 (30.4%)	32 (69.6%)	0.65	0.31–1.4	0.273
Health status worsened during the pandemic					
No	339 (16.8%)	1674 (83.2%)			ref
Yes	6 (11.5%)	46 (88.5%)	1.03	0.4–2.63	0.954
Mental health worsened during the pandemic					
No	321 (16.8%)	1589 (83.2%)			
Yes	24 (15.5%)	131 (84.5%)	0.62	0.36–1.08	0.091

* Univariate analysis adjusted by age for a 3–15-year age group, using a binomial logistic regression with all 20 imputed datasets, pooling the results through Rubin’s Rules (RR).

** Significative variables (p<0.050)

A positive association was found between COVID-19 vaccine acceptability and when father (OR 2.45 95%CI 1.46–4.1) or mother (OR 1.68 95%CI 1.14–2.47) were employed, living with a healthcare work (OR 1.52 95%CI 1.03–2.25), a good perception about their current health status (OR 4.68 95%CI 1.16–18.84) and, adherence to vaccination strategies (OR 18.8 95%CI 6.93–50.98) and vaccination routine behavior (OR 2.7 95%CI1.08–6.75) (Table 4).

In multivariate analyses, the variables associated with vaccine acceptability were age, adherence to previous vaccination, father work situation, influence of health care workers. Variables associated with refusal or hesitancy was use of herbal supplements and homeopathies (Table 5).

**Table 5:** Multivariate analysis between sociodemographic, perceptions, attitudes, behavior and knowledge indicators among parents of under 16 years students, in SSNC. Catalonia, January 2022.

Variable	Multivariate analysis*		
	OR	95%CI	p-value
Age group 6–11 years	3.39	2.53–4.53	<0.001
Age group 12–15 years	31.39	18.98–51.92	<0.001
Adherence to vaccination campaigns and strategies	17.92	6.57–48.87	<0.001
Use of herbal supplements or homeopathies	0.47	0.31–0.74	0.001
Father working status: employed	2.42	1.42–4.12	0.001
Living with health care worker (HCW)	1.51	1–2.27	0.051

* Multivariate analysis by a Deletion Substitution Addition (DSA) machine learning algorithm with 50 iterations. The performance of the models was accessed by the Akaike Information Criterion (AIC), adjusted R-square, criteria that defined the choice for the DSA model.

The AIC for the DSA model was 1504.17 (SD = 11.47), mean of the 20 imputed data sets, and the adjusted mean R-square was 0.2 (SD = 0.006), which corresponds to the variability of the R-square obtained by the models of each of the imputed data sets, and suggests that similar results were obtained across all imputed datasets and may be an indicator of a high-quality imputation process.

## Discussion

Our study found the COVID-19 vaccine acceptability was high among adolescents (96.7%) and parents (83.3%). The main reason to refusal and hesitancy reported by parents was concern about side effects, safety and lack of information, all variables associated. On the other hand, variables with impact in the vaccine acceptability were, students age, compliance of routine vaccination, work situation, use of alternative practices as homeopathy, perception about current health, and COVID-19 related impacts.

The prevalence of COVID-19 vaccine hesitancy in Spain was estimated at 6.2%, the lowest according to a study with unvaccinated participants from eight Europeans countries, proceeded in June 2021, and the main determinants are gender, age, education and employment [11]. A meta-analysis estimates the prevalence of COVID-19 vaccine hesitancy at 25% globally, recommending interventions among women, people with low educational levels, unemployed, people living in households with five or more individuals, and those who no trust in the COVID-19 safety [5].

A review found a global average for COVID-19 vaccine acceptance of 61.4%, with significantly difference between countries, variating between 42.9% in Saudi Arabia and 91.4% in Brazil [17], while the overall Europe acceptance was 72.6% [11]. Another study found that in United States 49% of parents were agreeing to vaccinate their children and 44% are planning to vaccinate them when the vaccine become available [28]. In Saudi Arabia, 47.6% of parents were ready to vaccinate their children [29], in Turkey, 36% of parents were willing to their children receive the vaccine [30], however 66% of parents were hesitant about a foreign vaccine and 37.4% were hesitant with a domestic vaccine [31].

In this study, the highest rates of refusal of the COVID-19 vaccine were among parents of students aged 3 to 4 years (41–49%), however, at the time of the study, there was still no vaccine approved for this population. For the 5–11 group, the vaccine had been approved during the first round of data collection, and although we found 30–22% refusal, we also found a significant increase in vaccine coverage (0–63%) immediately after approval of the vaccine for this age group. Similar behavior in relation to vaccine refusal rates was observed [32], where besides the data collection about vaccination have been carried out in the recent approval context, the vaccination schedule was just starting, and the services were still organizing for this demand. The contradictory arguments about the risk-benefit and severity of infection in this age group probably also contributed to this hesitation. Finally, we found that parents or guardians of older children were more likely to allow their vaccination, a result similar was found in another study [23].

The main reason to refusal and hesitancy reported by parents was concern about side effects, safety and lack of information, all variables associated. Variables with impact in the vaccine acceptability were, students age, compliance of routine vaccination, work situation, use of alternative practices as homeopathy, perception about current health, and COVID-19 related impacts.

Socioeconomic characteristics can be an obstacle to vaccination strategies, even considering the free vaccine [3, 33], for example, in our study we found that being employed was associated with a higher probability of accepting the vaccination of their children, while a greater number of people living in the same household, a decrease in the family’s economic situation during the pandemic was associated with a lower probability to vaccinate their children. Social determinants and characteristics related to less access to health policies and information had a greater impact on vaccine acceptability, a result also observed in different studies on coverage and acceptability among low-, middle- and high-income countries [4, 34]. Moreover, it is known that the burden of COVID-19 has affected different socioeconomic groups with different intensity, as observed in this populational study about mortality associated with socioeconomic status in Barcelona, Spain [35].

The parents’ migratory situation, when at least one was born abroad but lives in Spain, was associated with vaccine hesitancy, a similar result was founded in a Canadian study [18]. Disparities in vaccination rates among students of different ethnicities was also demonstrated previously [15].

As expected, parents who live with health professionals were more likely to vaccinate their children. This result reinforces the important role of health care professionals on vaccine promotion strategies. Vaccine hesitancy among health professionals, can be a very important problem to public health strategies and, in this case, for vaccine acceptance, since trust in public health authorities can be an important influence on the decision to vaccinate or vaccinate persons in their care [3, 36], as well as the message that reinforces safety and efficacy, disseminated by health professionals when vaccinating, can be useful to minimize vaccine hesitancy and refusal [34]. Also, they are a high-risk group for exposure to SARS-CoV-2 and had an understandable role in the transmission of the virus, for this reason, HCW were a priority group eligible for vaccination as soon as possible [36, 37].

Parents who had perceived their children are healthy were more likely to allow them to be vaccinated, the same result was observed in a study with health professionals [38]. The history of receiving previous vaccine for another diseases among parents and students had increases the likely to be vaccinated and allow vaccinated their children, also, parents and adolescents who were previous vaccinated had more likely to accept the COVID-19 vaccine, findings consistent with another previous studies [38–43].

Use of alternative medicines such as homeopathy, that had a negative association with vaccine acceptability among both, parents and adolescents. This can be related to the lower use of health services and distrust of pharmacological therapies by these users. In addition, personal beliefs were already associated with vaccine refusal among parents in previous studies [44]. A Spanish study found a lower demand for influenza vaccination among homeopathy users, that rejects the vaccine for themself and for their children [45]. Moreover, family uptake behavior, can affect vaccine uptake among adolescents, as indicated in previous studies [39].

For unvaccinated students, the main reasons to vaccine refusal or hesitancy were concern about side effects, safety and vaccine distrust, agreeing with other studies in similar populations that demonstrated the significance of these variables in the decision not to be vaccinated [40, 41]. The associated variables among students were risk perception, knowledge, alternative medical practices as homeopathy and compliance of routine vaccination. Students with higher perceived knowledge about COVID-19 were more likely to accept the vaccine, that is coherent with the discussion about access to adequate health information as an important predictor to vaccine acceptability [46–48].

Students that belief is easy to avoid a SARS-COV-2 infection, that is, who do not perceive the risk to be infected were more likely to refuse the vaccine against COVID-19, consistent with other studies [40, 49].

A previous SARS-CoV-2 infection as a reason for vaccine refusal or hesitation increased between October 2021 and January 2022 among the participants may be related with the large wave caused by the Omicron variant in Catalonia, occurred in early December 2021, between the two points of the study data collection. The higher transmissibility and increasing infections, in fully vaccinated people included, could be associated with untrust and doubts about the vaccine effectiveness increasing the hesitation. Moreover, unlike the beginning of the pandemic, the perception of risk has changed, and people believe that COVID-19 has become a mild disease now.

Vaccine access is an important goal for health policies. It is very important understand how the lack of information, personal beliefs, limited health literacy and lower risk awareness was associated with vaccine refusal and hesitancy as discussed by another research [40]. Previous study showed that recommendation for the Government has strongly associated with acceptance [48], and especially when qualified information is available [23, 29, 44, 50, 51].

## Limitations

Although the study design was adequate for the purpose of the study, the results should be extrapolated with caution. Predictive capacity of the statistical models applied in this study are considerable high, but, despite the high prevalence of COVID-19 vaccine acceptability in our sample, the study population does not represent the overall children and parents of the country.

The sample heterogeneity allows to generate several hypotheses, these would eventually need to be confirmed with population-based surveys. The reported vaccine acceptability may not reflect actual vaccination behavior as well. The fact that vaccination intentions were only asked for those who were not yet vaccinated, have also reduced this population size over time. Therefore, new research, especially longitudinal studies at different intervals, will be needed to investigate and evaluate this behavior in different periods of COVID-19 vaccination strategies.

## Conclusions

This is the first investigation into COVID-19 vaccine hesitancy among parents and adolescents in Catalonia, Spain. Precaution regarding parental vaccination of children is consistently present across different periods of the pandemic, suggesting that individual risk-benefit continues to guide decision-making processes regarding vaccination.

Findings on the impact of social and structural factors on COVID-19 vaccine acceptability highlight the importance of specifically targeted interventions to address frequent misunderstandings and reinforce the common benefit of vaccination at the population level.

The evolving nature of SARS-CoV-2 and the potential role of vaccines in the pediatric population are still full of uncertainties. Both understanding the biological evolution of the virus and the persistence of natural and acquired immune protection will be crucial in defining vaccine recommendations for children.

Finally, the emergency response to SARS-CoV-2 needs to leave us a legacy of a health system prepared for increasingly efficient responses to future threats to public health. In this context, monitoring compliance and evolution of preventive public health measures, such vaccine hesitancy has been important to better understand the interaction between different multilevel determinants and its maintenance over time will be necessary to correctly delineate future interventions.
